# Knowledge, Attitudes, and Awareness of Food and Drug Interactions (FDI) Among Nurses on General Medical Wards: A Cross-Sectional Study

**DOI:** 10.1177/23779608241280847

**Published:** 2024-09-27

**Authors:** Reem Elajez, Raja Alkhawaja, Vahe Kehyayan, Khadija AL Shukaili, Esmat Swallmeh

**Affiliations:** 1 Pharmacy Department, Hamad General Hospital, 36977 Hamad Medical Corporation, Doha, Qatar; 2 61781 University of Doha for Science & Technology, Doha, Qatar; 3 36977 Hamad Medical Corporation, Doha, Qatar; 4 Calgary University, Doha, Qatar

**Keywords:** food–drug interaction, nurses’ knowledge, patient safety

## Abstract

**Background:**

Food and nutritional supplements can interact with medication and cause drug interaction. Registered nurses play an essential role in patients’ safety related to drug interaction.

**Objective:**

Explore nurses’ knowledge, attitudes, and awareness regarding food–drug interactions (FDIs).

**Method:**

A cross-sectional survey was conducted among nurses working in the general medical wards at Hamad General Hospital (HGH) in Qatar. The survey questionnaire comprised 50 multiple-choice questions, encompassing three key sections: (a) demographic details, (b) assessment of FDIs knowledge, and (c) exploration of beliefs, attitudes, and practices concerning FDIs.

**Results:**

A total of 227 surveys were collected. Nurses’ average score for FDI knowledge across all responses was 20 out of 34 (IQR 16–25). Notably, there were no statistically significant differences in average scores based on respondents’ demographic subgroups. While nurses displayed a high level of knowledge (over 90%) regarding the timing of food and drug intake for proton pump inhibitors, nonsteroidal anti-inflammatory drugs, and thyroid hormones, they exhibited limited awareness of the possible FDIs related to carvedilol and furosemide. Approximately half of the participating nurses (56.4%) rated their FDI knowledge as satisfactory. However, only 42.3% claimed to be always educating patients about FDIs in their daily practice. Two-thirds (67.4%) of respondents believed educating patients about FDIs should primarily fall under the responsibility of pharmacists.

**Conclusion:**

This study highlights the nurses’ knowledge, attitudes, and beliefs of FDIs. Prompt intervention is required to enhance the nurse's awareness and knowledge in this domain which may impact patient care and drug safety.

## Introduction

Food–drug interactions (FDIs) arise when specific nutrients present in foods interact with drugs when consumed concurrently (Yaheya & Ismail, 2009). According to the Food and Drug Administration (FDA), FDI is defined as “a situation where a food affects the activity of a drug; for instance, the effects are increased or decreased, or a new effect of that drug is produced that would not occur without the consumption of that food” (Owens & Toone, 2014). The risks associated with FDIs can be influenced by a range of factors such as age, gender, medical conditions, body composition, nutritional status, and the concurrent use of multiple medications (Murphy et al., 2005).

Food-drug interactions represent a significant facet of drug interactions that warrant attention (Ased et al., 2018; Bushra et al., 2011; Pennington & Frandsen, 2014). Despite research indicating that reducing drug-drug interactions by 1% could result in an annual savings of $29,250 in indirect costs (Kwan & Brodie, 2001), research on FDIs is scarcer (Bushra et al., 2011).

Numerous studies confirm the crucial role of registered nurses in ensuring patient safety related to the prevention of FDIs. Nurses undertake vital tasks such as medication scheduling, administration, and monitoring (Institute of Medicine, 2000, 2011), which necessitate a comprehensive understanding of FDIs to prevent adverse incidents (Faria & Cassiani, 2011). The Joint Commission on Accreditation of Healthcare Organizations (JCAHO) advocates nurses to actively counsel patients about FDIs (Enwerem, 2017), including advising on diet modifications while taking specific medications (Abbasi Nazari et al., 2011), and understanding whether drugs require ingestion with or without food (Benni et al., 2012).

## Review of Literature

Few studies have assessed nurses’ competence regarding FDIs. In one study, nurses in general wards achieved a mean knowledge score of 10.34 ± 11.15 out of 30 on FDIs (Moradi et al., 2016). Multiple researches have demonstrated that about half of the nurses studied had inadequate FDI knowledge, scoring between 44% and 65% (Enwerem, 2017; Enwerem & Okunji, 2015, 2016; Lafi et al., 2019; Sajid et al., 2017). A logistic regression analysis predicting factors influencing healthcare professionals’ FDI knowledge showed that occupation was the only significant factor. Compared to doctors, pharmacists, and dietitians, nurses exhibited lower FDI knowledge (Osuala et al., 2021). Another study comparing FDI knowledge among different healthcare professionals found nurses to have the lowest scores (Degefu et al., 2022). Despite the significance of FDIs on patients’ health outcomes, evidence about nurses’ knowledge and practice regarding FDI in Qatar is lacking.

Aligned with the objective of enhancing patient care and drug therapy quality, ensuring patient safety, mitigating complications, and curtailing treatment costs, this study was conducted to explore the knowledge of FDIs among registered nurses’ working on the general medical wards of the Hamad General Hospital (HGH) in Qatar.

## Methods

### Design and Participants

The study was a descriptive observational cross-sectional survey. Data collection was carried out within the general medical wards of Hamad General Hospital (HGH), the largest tertiary care hospital in Qatar. This study included all registered nurses in the medical wards at HGH with the exception of head nurses and in-charge nurses who had previously been involved in assessing the survey questionnaire's validity and reliability. No other inclusion/exclusion criteria were used To achieve a 95% confidence level with a 5% margin of error, the sample size of at least 206 nurses is needed. However, a 10% increase in the sample size (i.e., 227) was initially considered to accommodate non-responders.

### Instrument

The research team constructed a questionnaire based on a critical review of the relevant literature and similar prior studies, employing resources such as LEXICOMP, MICROMEDEX, and the Nursing 2018 Drug Handbook as reference guides. The questionnaire included 50 multiple-choice questions distributed across three primary sections: (a) demographic information, (b) general and specific knowledge pertaining to FDIs, and (c) beliefs, attitudes, and practices concerning FDIs. The assessment of nurses’ knowledge relied solely on the second section of the survey, which comprised 34 questions pertaining to their knowledge of FDIs. These questions were presented in formats of “yes, no, I don't know” or “before/with the meal, after the meal, I don't know.” Correct responses were given a score of 1, while incorrect or missed answers received a score of 0. The evaluation of beliefs, attitudes, and practices consisted of 11 questions designed as multiple-choice or binary “yes, no” responses.

### Survey Validity and Reliability

The survey questionnaire's content validity was established through a review of pharmacology textbooks. Three authors independently examined the questionnaire to ensure the accuracy and relevance of the questions with respect to the study's objectives and medication administration practices within the hospital. To finalize its validity, a pilot study was conducted involving ten head and in-charge nurses from the eligible participating wards. During the pilot study, these participants were asked to provide open feedback on the content, the time required for questionnaire completion, and the potential for maintaining anonymity. The estimated time needed to complete the survey was determined to be between 8 to 10 min.

Questionnaire reliability was then ascertained through another pilot test involving 20 nurses working at HGH, sharing similar characteristics to the study participants. The data generated from this second pilot was excluded from the final sample and analyses. The analyses of collected pilot data yielded a reliability coefficient (alpha) of .85, attesting to the questionnaire's reliability.

### Data Collection

An email invitation was sent to all registered nurses working in the general medical wards at HGH, comprising an estimated count of 440 nurses. This email included a brief description of the study and its purpose, along with a direct web link to the questionnaire. By submitting the survey through this link, participants were considered to have given their consent. Survey Monkey was the used platform for data collection.

Throughout the data collection period, the authors faced several challenges, including missing answers for some questions. These were addressed promptly by making such questions mandatory. This adjustment ensured that all required information was collected from each respondent, thereby improving the completeness and reliability of the data.

To encourage participation, several reminders were sent at regular intervals. These reminders were personalized and tailored to address potential concerns and highlight the importance of the participants’ input.

### Statistical Analyses

The statistical analysis was done using Statistical Package for Social Sciences by IBM Incorporated (SPSS INC. Chicago, IL) version 19.0. Descriptive analysis was used to present the nurses’ knowledge of FDIs. Results were expressed mainly as mean ± standard deviation (SD) and/ or percentages. Percentages of correct responses (i.e., score) were calculated by dividing the number of correct responses by the total number of responses received at the level of individual questions. To explore potential associations between demographic factors and knowledge levels, the chi-square test and Fisher's exact test were applied as applicable. For continuous data, such as knowledge score, the Mann–Whitney test or the Kruskal–Wallis test was employed as deemed suitable. All presented *p*-values were two-tailed, and significance was established at *p*-values less than .05.

### Ethical Consideration

The study was executed in accordance with the stipulations of the Institutional Review Board/Human Subjects Research Committee of the Medical Research Center, Hamad Medical Corporation, Qatar (MRC-01-18-448) and obtained the necessary approval. Participants’ responses remained anonymous and solely used for research purposes without any associated risk resulting from involvement in the study.

## Results

A total of 227 responses were collected for the purposes of this study, reflecting a response rate of 51.6% (227/440). Majority of respondents were female 177 (78%) and most were aged 25–35 years. In terms of education, 103 (45.4%) respondents indicated having attained a postgraduate or master's degree, while 84 (37%) held diplomas. Participants primarily graduated from the Philippines and India. A detailed description of the participants’ characteristics is outlined in Table 1.

**Table 1.** Participants’ Demographic Characteristics.

	Number (percentage %)
Gender	
Male	50 (22%)
Female	177 (78%)
Age	
Less than 25 years	1 (0.5%)
25–35 years	154 (67.8%)
36–45 years	57 (25.1%)
More than 45 years	15 (6.6%)
Highest education level	
Diploma	84 (37%)
Undergraduate	40 (17.6%)
Postgraduate /master's degree	103 (45.4%)
Country of graduation	
Philippine	125 (55.1%)
India	83 (36.5%)
Egypt	5 (2.2%)
Jordan	9 (4%)
Others	5 (2.2%)
Years of nursing experience:	
Less than 5 years	41 (18%)
5–10 years	114 (50.3%)
11–15 years	49 (21.6%)
More than 15 years	23 (10.1%)

The evaluation of participants’ knowledge regarding FDIs was based on their accuracy in responding to the 34 knowledge-related questions in Section 2 of the questionnaire (Table 2). The collective mean score for all responses stood at 20 out of 34 (with an Interquartile Range (IQR) of 16–25), and the highest achieved score was 32 out of 34. Given that not all participants answered all questions, the total knowledge score was adjusted to encompass only those who had provided complete answers in Section 2 (*n* = 193). Consequently, the adjusted average score was determined to be 22 (with an IQR of 19–26). There were no statistically significant differences in average scores among respondents’ demographic subgroups. Factors such as age, years of experience, educational degree, and country of graduation were not found to exert any influence on the knowledge score (Supplementary Table A).

**Table 2.** Knowledge and Awareness of Food-Drug Interactions.

Q.#	Question	Number of correct answers	Total number of responses*	Percentage (%) of correct answers**
*General FDI knowledge*				
1	FDI includes drug interacting with □ Diet □ Iron/ Vitamin supplements □ Alcohol and fruit juices □ All of the above	119	227	87.7
2	Food can speed up or slow down the action of a drug □ Yes □ No	222	227	97.8
3	At which pharmacokinetic level do foods most commonly interfere with drugs? □ Absorption □ Distribution □ Metabolism □ Excretion	198	227	87.2
*FDI knowledge at the level of individual drugs and the correct timing of food and drug intake*				
4	Avoid taking milk and iron rich food with fluoroquinolones	136	196	69.4
5	Chicken liver, aged cheese should be avoided with some medication like linezolid	124	200	62
6	Acidic foods/beverages (tomato sauce, and citrus juices) can be taken with antibiotic	115	196	58.7
7	If patient is taking tetracycline, he/she should avoid milk and dairy products	143	195	73.3
8	Both itraconazole capsule and suspension should be taken after meal	59	194	30.4
9	Fatty diet increase absorption of hydrochlorothiazide and albendazole	70	195	35.9
10	High fiber meal could enhance absorption of digoxin	77	194	39.7
11	Garlic can enhance the effect of antihypertensive medication	131	196	66.8
12	A patient on Lisinopril should be encouraged to eat more banana	92	192	47.9
13	Carvedilol must be taken on empty stomach	16	196	8.2
14	Spironolactone must be avoided with potassium rich foods	132	193	68.4
15	Cranberry juice can decrease effect of warfarin	54	195	27.7
16	Patients on Warfarin should avoid foods like Spinach, broccoli in large quantities	142	194	73.2
17	Patient on NSAIDs should consume large amount of tea, coffee & chocolates	153	196	78.1
18	Caffeine increases the risk of theophylline drug toxicity (like nervousness and tremor)	157	193	81.3
19	Patient on L-thyroxin must avoid turnips, cauliflower, and cabbage	115	194	59.3
20	Grapefruit interacts with different medications and may produce lethal side effect	128	192	66.7
21	Meals rich in protein and fat can enhance levodopa absorption	60	194	30.9
22	Esomeprazole absorption could be increased with fatty meals	156	197	79.2
23	L-thyroxin requires the interruption of diet through feeding tube for 1-2 h	147	196	75
24	Phenytoin requires the interruption of diet through feeding tube for 1-2 h	114	199	57.3
25	Alendronate must be taken on empty stomach	115	208	55.3
26	Lansoprazole should be taken …………. meal	214	225	95.1
27	Glipizide, Rifafour® should be taken ………. meal	172	226	76.1
28	NSAIDs, steroids are advised to be taken………. meal	206	226	91.2
29	Rivaroxaban dose ≥15 mg must be taken ………. meal	152	225	67.6
30	Thyroid hormones (levothyroxine) should be taken ………. Meal	217	226	96
31	Metformin, gliclazide M/R should be taken ……. meal	173	226	76.5
32	Genvoya (Elvitegravir/cobicistat/tenofovir/emtricitabine) should be taken………..…. meal	155	224	69.2
33	Furosemide should be taken ………. meal	51	226	22.6
34	Cinacalcet should be taken …………. meal	196	226	86.7

* Not all questions had 227 responses, as some questions were left empty.

** Percentages were calculated by dividing the number of correct responses by the total number of responses received.

Concerning general FDI knowledge (questions Q1-3), the majority of participants provided accurate answers to these questions. However, in FDI knowledge related to individual drugs (questions Q4-26), respondents demonstrated lower proficiency, particularly in questions addressing the influence of specific foods on drug absorption. Notably, inquiries about the impact of protein/fat-rich meals on levodopa, fatty diets on hydrochlorothiazide and albendazole, and high-fiber meals on digoxin were correctly answered by only 30.9%, 35.9%, and 39.7% of nurses, respectively. In relation to the impact of drug formulation on FDIs, only 30.4% of respondents accurately recognized the difference between itraconazole capsule and suspension regarding the optimal timing for administration in relation to meals.

Regarding the appropriate timings of food and drug intake, the majority of participants (over 90%) exhibited the right comprehension of proton pump inhibitors (e.g., lansoprazole), nonsteroidal anti-inflammatory drugs (NSAIDs), steroids, and thyroid hormones (i.e., levothyroxine). However, their awareness of the appropriate schedule of furosemide in relation to mealtimes was notably deficient.

Regarding participants’ beliefs and attitudes, the majority (56.4%) evaluated their knowledge of FDIs as satisfactory, while 15.4% rated their knowledge as poor. Despite the alignment between the mean knowledge score and the self-rated knowledge category (i.e., both Excellent and Very good had a score of 22, Satisfactory had a score of 21, and Poor had a score of 17), a similar score between “Excellent” and “Very good” of 22 along with the slight difference with “Satisfactory” of 21 suggests a discernible incongruence between self-perceived proficiency and actual performance. A significant proportion of participants 130 (57.3%) stated that they had not encountered any occasions of FDIs in their clinical practice. Nearly all participants (97.8%) expressed the necessity of acquiring more knowledge about FDIs, and 99.1% held the belief that each patient should receive counseling about FDIs. However, the practice of educating patients about FDIs was claimed to be always done by only 96 participants (42.3%) as part of their daily routines. Approximately two-thirds (67.4%) of respondents attributed the responsibility of educating patients about FDIs to pharmacists, and 54.6% had consulted pharmacists regarding FDIs (Table 3).

**Table 3.** Beliefs, Attitudes, and Practices of Nurses About FDI.

	Number	Percentage (%)
Have you encountered any FDI before		
Yes	97	42.7
No	130	57.3
Do you believe it's necessary to know about FDI		
Yes	222	97.8
No	5	2.2
Do you have any system to identify FDI in the workplace		
Yes	150	66.1
No	77	33.9
How do you rate your knowledge about FDI		
Excellent	10	4.4
Very good	54	23.8
Satisfactory	128	56.4
Poor	35	15.4
Are you considering food interactions when scheduling the drug		
Yes	219	96.5
No	8	3.5
Do you believe that each patient needs counseling about FDI		
Yes	225	99.1
No	2	0.9
How often do you educate patients about FDI		
Always	96	42.3
Sometimes	125	55.1
Never	6	2.6
Who do you believe should educate patient about FDI		
Nurse	36	15.9
Pharmacist	153	67.4
Dietician	32	14.1
Doctor	6	2.6
Have you ever consulted pharmacist about FDI		
Yes	124	54.6
No	103	45.4
What is your source of information if you want to check about FDI (choose all apply)		
Pharmacist	176	77.5
Dietician	105	46.3
Drug leaflet	152	67
Online search engine	145	63.9
Another nurse	75	33
Book	62	27.3
Drug database	123	54.2
How awareness about FDI can be improved MOSTLY among nurses		
Conferences/seminars	72	31.7
Workshops	54	23.8
Online courses	78	34.4
Trusted references	20	8.8
Other	3	1.3

Seventy-seven participating nurses claimed the absence of any established system to identify FDIs in their workplace, despite the actual existence of such resources. Yet, the most frequent sources of information cited by participants were pharmacists, drug leaflets, and online search engines, with corresponding proportions of 77.5%, 67%, and 63.9%, respectively. Nurses also expressed the belief that the most efficacious methods for enhancing nurses’ awareness about FDIs would include enrolling in online courses, as well as participating in conferences, seminars, or workshops.

## Discussion

Despite the serious clinical implications of FDIs, this type of interaction is generally overlooked as a form of drug interaction, especially among nurses who could potentially have a crucial role in its prevention. The study's findings revealed that nurses working in medical wards at HGH exhibited an average level of knowledge regarding FDIs. Notably, their knowledge was found to be unaffected by variables such as age, years of experience, educational degree, or country of graduation. Although length of experience is often presumed to influence knowledge, in this study the finding was in line with previous studies, in which the length of experience did not exert a discernible impact on FDI knowledge levels (Enwerem & Okunji, 2015; Ives et al., 1996). This could be explained by the recent emphasis on FDI topics in nursing curricula which could potentially equalize knowledge across nurses of varying experience levels (Enwerem, 2017). Additionally, nurses might actively share their FDI insights with newer colleagues, contributing to a common understanding. Contrasting findings however were still observed in other studies, revealing noteworthy differences in knowledge levels among nurses with different degrees of experience (Moradi et al., 2016; Osuala & Ojewole, 2021).

In this study, nurses demonstrated a good understanding of fundamental FDI concepts. They exhibited awareness of the fact that food can either accelerate or decelerate the effects of a medication. Additionally, they displayed knowledge of the pharmacokinetic stages at which food most frequently interacts with drugs, as well as the various types or categories of food that fall within the realm of food-drug interactions. Conversely, an unfortunate lack of knowledge was observed when evaluating responses to statements concerning the interaction of specific foods with drugs, and knowledge of the timing of food intake in relation to drug administration. A notable deficiency in knowledge, with scores falling below 50%, was evident in the realm of cardiovascular medications, despite their frequent use. For instance, low scores were observed in questions concerning medications like digoxin, carvedilol, hydrochlorothiazide, and furosemide. This knowledge gap may be attributed to the fact that these medications lack widely recognized FDI information, despite their clinical significance, unlike other more well-documented drugs (Grześk et al., 2021; Vuong et al., 2023). Another probable explanation could be that while nurses possess a general understanding of the concept of interactions between certain foods and drugs, such as the need to limit potassium intake with angiotensin receptor blockers (ARBs), or the influence of vitamin K-rich foods on warfarin, they may struggle to directly apply this knowledge in practical scenarios to identify whether certain foods are rich in potassium or vitamin K. This became evident when questions were posed about the use of warfarin with foods like spinach and broccoli, which are well-known for their high vitamin K content; responses were correct in over 70% of cases. However, when the same question was asked about warfarin and cranberries, which are exceptionally rich in vitamin K but not as widely recognized for this characteristic, correct answers dropped to less than 30%.

On the other hand, nurses have demonstrated a better level of knowledge regarding FDI concerning frequently prescribed antimicrobials. For instance, approximately two-thirds of the nurses were able to adequately answer questions pertaining to the use of milk and iron-rich foods in combination with antibiotics like tetracycline and fluoroquinolones. Similarly, nurses were well-informed about antiretroviral medication (e.g., Genvoya®) and its optimal administration with food. Additionally, despite linezolid not typically falling under the monoamine oxidase inhibitors (MAOIs) classification, nurses exhibited a satisfactory understanding of the potential risks associated with consuming tyramine-containing foods while using linezolid given that linezolid can also inhibit MAO. However, when it came to less commonly utilized antimicrobials such as itraconazole and albendazole, nurses displayed a deficit in knowledge. Nurses may have limited familiarity and experience with these specific drugs, as they are more commonly prescribed for outpatient rather than inpatient care, which could explain their difficulty in providing accurate responses to related questions.

In addition to the knowledge assessment, this study highlighted the robust beliefs held by nurses regarding the pivotal role of pharmacists in managing FDIs, as they are considered the primary authority responsible for counseling patients and serving as a central source of expertise for addressing any questions or concerns about FDIs. In Qatar, clinical pharmacists collaborate closely with nurses, physicians, and other healthcare providers, addressing their concerns, offering medication therapy management, and providing patient counseling, including aspects related to FDI. These outcomes align with the findings presented by both Nazari and Moradi et al. who similarly highlighted the influential role of clinical pharmacists in instructing nurses to mitigate the occurrence of FDIs (Abbasi Nazari et al., 2011; Moradi et al., 2016).

## Strengths and Limitations

This study exhibited several notable strengths. Most notably, it marks the pioneering effort to assess nurses’ knowledge and attitudes pertaining to FDI in Qatar. Furthermore, the study was conducted within the principal and largest tertiary hospital in the nation. The study's robustness was enhanced by using a validated questionnaire that comprehensively covered various facets of FDIs. Yet, this study had several limitations worth mentioning. First, because of its cross-sectional design, offering only a singular snapshot of nurses’ knowledge at a specific timepoint, the results could not be generalizable to all registered nurses in all other tertiary hospitals in Qatar. Additionally, the presence of some missing answers posed a limitation, potentially impacting the robustness of analyses and the interpretation of the results. Furthermore, the data collected were confined to medical wards within HGH, necessitating caution when extrapolating the study's conclusions to other divisions or departments within HGH and/or HMC. Nevertheless, the validated questionnaire employed in this study holds the potential for wider applicability, enabling its use to assess nurses’ knowledge and practices regarding FDIs across various settings and possibly even on an international scale.

## Implications for Practice

Given the existing level of FDI knowledge and practices among nurses, it is advisable to intensify initiatives for conducting specialized training workshops on FDIs. Policymakers should actively work towards augmenting nurses’ expertise in this field through collaborative efforts with the hospital's pharmacy department and nursing programs at universities. It is essential for nurses to recognize their responsibility in averting such interactions and incorporate this awareness into their daily routines, either by updating their job descriptions or integrating it into practice requirements. The insights from the study can form the basis for developing interventions designed to improve nurses’ comprehension and consciousness of FDIs.

## Conclusion

This study sheds light on the current registered nurses’ knowledge, attitudes, and beliefs about FDIs within medical wards at HGH in Qatar. Results highlight the existence of knowledge gaps among nurses and underline the imperative to improve their understanding and awareness of this subject, as well as their role in preventing such interactions. Dedicated and coordinated efforts to create interventions that prioritize the improvement of registered nurses’ comprehension and awareness regarding FDIs are highly required.

## Supplemental Material

sj-docx-1-son-10.1177_23779608241280847 - Supplemental material for Knowledge, Attitudes, and Awareness of Food and Drug Interactions (FDI) Among Nurses on General Medical Wards: A Cross-Sectional Study Supplemental material, sj-docx-1-son-10.1177_23779608241280847 for Knowledge, Attitudes, and Awareness of Food and Drug Interactions (FDI) Among Nurses on General Medical Wards: A Cross-Sectional Study by Reem Elajez, Raja Alkhawaja, Vahe Kehyayan, Khadija AL Shukaili and Esmat Swallmeh in SAGE Open Nursing

sj-docx-2-son-10.1177_23779608241280847 - Supplemental material for Knowledge, Attitudes, and Awareness of Food and Drug Interactions (FDI) Among Nurses on General Medical Wards: A Cross-Sectional Study Supplemental material, sj-docx-2-son-10.1177_23779608241280847 for Knowledge, Attitudes, and Awareness of Food and Drug Interactions (FDI) Among Nurses on General Medical Wards: A Cross-Sectional Study by Reem Elajez, Raja Alkhawaja, Vahe Kehyayan, Khadija AL Shukaili and Esmat Swallmeh in SAGE Open Nursing

sj-docx-3-son-10.1177_23779608241280847 - Supplemental material for Knowledge, Attitudes, and Awareness of Food and Drug Interactions (FDI) Among Nurses on General Medical Wards: A Cross-Sectional Study Supplemental material, sj-docx-3-son-10.1177_23779608241280847 for Knowledge, Attitudes, and Awareness of Food and Drug Interactions (FDI) Among Nurses on General Medical Wards: A Cross-Sectional Study by Reem Elajez, Raja Alkhawaja, Vahe Kehyayan, Khadija AL Shukaili and Esmat Swallmeh in SAGE Open Nursing
