# Supplementary material for: Knowledge, Attitudes, and Awareness of Food and Drug Interactions (FDI) Among Nurses on General Medical Wards: A Cross-Sectional Study
Source: SAGE Open Nurs. 2024 Sep 27;10:23779608241280847. doi: 10.1177/23779608241280847 (PMC11475285; doi:10.1177/23779608241280847)
Supplement: sj-docx-1-son-10.1177_23779608241280847 - Supplemental material for Knowledge, Attitudes, and Awareness of Food and Drug Interactions (FDI) Among Nurses on General Medical Wards: A Cross-Sectional Study [file sj-docx-1-son-10.1177_23779608241280847.docx]

**Supplementary Table-A: Knowledge scores among respondents' demographic subgroups**

|  | **Average Score** | **P-value** |
| --- | --- | --- |
| **Gender** | | **0.216** |
| Male | 20.8 |  |
| Female | 21.3 |  |
| **Age** | | **0.160** |
| Less than 25 years | 20.5 |  |
| 25-35 years | 21.2 |  |
| 36-45 years | 20.4 |  |
| More than 45 years | 22.2 |  |
| **Highest education level** | | **0.708** |
| Diploma | 21.4 |  |
| Undergraduate | 21 |  |
| Postgraduate /master’s degree | 21.1 |  |
| **Country of graduation** | | **0.098** |
| Philippine | 21.4 |  |
| India | 21.4 |  |
| Egypt | 21 |  |
| Jordan | 18 |  |
| Others | 20.5 |  |
| **Years of nursing experience:** | | **0.447** |
| Less than 5 years | 20.8 |  |
| 5-10 years | 20.9 |  |
| 11-15 years | 21.9 |  |
| More than 15 years | 22 |  |
