# Supplementary material for: Knowledge, Attitudes, and Awareness of Food and Drug Interactions (FDI) Among Nurses on General Medical Wards: A Cross-Sectional Study
Source: SAGE Open Nurs. 2024 Sep 27;10:23779608241280847. doi: 10.1177/23779608241280847 (PMC11475285; doi:10.1177/23779608241280847)
Supplement: sj-docx-3-son-10.1177_23779608241280847 - Supplemental material for Knowledge, Attitudes, and Awareness of Food and Drug Interactions (FDI) Among Nurses on General Medical Wards: A Cross-Sectional Study [file sj-docx-3-son-10.1177_23779608241280847.docx]

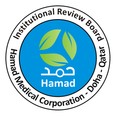
**Knowledge, Attitudes and Awareness of Food and Drug Interactions (FDI) among Nurses at General Medical Wards in Qatar**

| The aim of this survey is to assess the nurses’ knowledge & attitudes about Food and Drug Interactions (FDI). We would appreciate your time to complete the following survey. It should take **10 minutes** of your time.    Your responsive is voluntary and will be anonymous. Responses will not be identified by individual. All results will be combined together and analyzed as group. You can withdraw and decide not to complete the survey at any point. | | | | | | | |
| --- | --- | --- | --- | --- | --- | --- | --- |
| **Demographic information** | | | | | | | |
| **Gender:**   Male   Female | | **Age:**   Less than 25 years   25-35 years   More than 35 years | | **Your highest education level**   Diploma   Undergraduate   Postgraduate /master degree | | | |
| **Co**           | **untry of graduation**  Philippine  India  Egypt  Jordan  Others: ……………………………. | **Ye**         | **ars of experience:**  Less than 5 years  5-10 years  11-15 years  More than 15 years | **Current unit working at:**   5N1   5N3   3S3   AMAU | | | |
| **Knowledge about Food –Drug Int** | | **eract** | **ion (FDI)** |  | | | |
| ***General*** | |  |  |  | | | |
| **FDI includes drug interacting with**   Diet   Iron/ Vitamin supplements   Alcohol and fruit juices   All of the above | | **Food can speed up or slow down the action of a drug**   Yes   No | | **At what level do the foods interfere with the drug commonly**   Absorption   Distribution   Metabolism   Excretion | | | |
| ***Specific*** | | | | | | | |
| ***IDK:*** *I Don’t Know* | | | | | | | |
| Avoid taking milk and iron rich food with fluoroquinolones | | | | |  Yes |  No |  IDK |
| Chicken liver, aged cheese should be avoided with some medication like linezolid | | | | |  Yes |  No |  IDK |
| Acidic foods/beverages (tomato sauce, and citrus juices) can be taken with antibiotic | | | | |  Yes |  No |  IDK |
| If patient is taking tetracycline, he/she should avoid milk and dairy products | | | | |  Yes |  No |  IDK |
| Both itraconazole capsule and suspension should be taken after meal | | | | |  Yes |  No |  IDK |
| Fatty diet increase absorption of hydrochlorothiazide and albendazole | | | | |  Yes |  No |  IDK |
| High fiber meal could enhance absorption of digoxin | | | | |  Yes |  No |  IDK |
| Garlic can enhance the effect of antihypertensive medication | | | | |  Yes |  No |  IDK |
| A patient on Lisinopril should be encouraged to eat more banana | | | | |  Yes |  No |  IDK |
| Carvedilol must be taken on empty stomach | | | | |  Yes |  No |  IDK |
| Spironolactone must be avoided with potassium rich foods. | | | | |  Yes |  No |  IDK |
| Cranberry juice can decrease effect of warfarin | | | | |  Yes |  No |  IDK |
| Patients on Warfarin should avoid foods like Spinach, broccoli in large quantities | | | | |  Yes |  No |  IDK |

MRC-01-18-448 Validity: 16 06 2019 - 14 06 2020 E-stamped 20 Jun 2019

**Knowledge, Attitudes and Awareness of Food and Drug Interactions (FDI) among Nurses at General Medical Wards in Qatar**

| The aim of this survey is to assess the nurses’ knowledge & attitudes about Food and Drug Interactions (FDI). We would appreciate your time to complete the following survey. It should take **10 minutes** of your time.    Your responsive is voluntary and will be anonymous. Responses will not be identified by individual. All results will be combined together and analyzed as group. You can withdraw and decide not to complete the survey at any point. | | | | | | | |
| --- | --- | --- | --- | --- | --- | --- | --- |
| **Demographic information** | | | | | | | |
| **Gender:**   Male   Female | | **Age:**   Less than 25 years   25-35 years   More than 35 years | | **Your highest education level**   Diploma   Undergraduate   Postgraduate /master degree | | | |
| **Co**           | **untry of graduation**  Philippine  India  Egypt  Jordan  Others: ……………………………. | **Ye**         | **ars of experience:**  Less than 5 years  5-10 years  11-15 years  More than 15 years | **Current unit working at:**   5N1   5N3   3S3   AMAU | | | |
| **Knowledge about Food –Drug Int** | | **eract** | **ion (FDI)** |  | | | |
| ***General*** | |  |  |  | | | |
| **FDI includes drug interacting with**   Diet   Iron/ Vitamin supplements   Alcohol and fruit juices   All of the above | | **Food can speed up or slow down the action of a drug**   Yes   No | | **At what level do the foods interfere with the drug commonly**   Absorption   Distribution   Metabolism   Excretion | | | |
| ***Specific*** | | | | | | | |
| ***IDK:*** *I Don’t Know* | | | | | | | |
| Avoid taking milk and iron rich food with fluoroquinolones | | | | |  Yes |  No |  IDK |
| Chicken liver, aged cheese should be avoided with some medication like linezolid | | | | |  Yes |  No |  IDK |
| Acidic foods/beverages (tomato sauce, and citrus juices) can be taken with antibiotic | | | | |  Yes |  No |  IDK |
| If patient is taking tetracycline, he/she should avoid milk and dairy products | | | | |  Yes |  No |  IDK |
| Both itraconazole capsule and suspension should be taken after meal | | | | |  Yes |  No |  IDK |
| Fatty diet increase absorption of hydrochlorothiazide and albendazole | | | | |  Yes |  No |  IDK |
| High fiber meal could enhance absorption of digoxin | | | | |  Yes |  No |  IDK |
| Garlic can enhance the effect of antihypertensive medication | | | | |  Yes |  No |  IDK |
| A patient on Lisinopril should be encouraged to eat more banana | | | | |  Yes |  No |  IDK |
| Carvedilol must be taken on empty stomach | | | | |  Yes |  No |  IDK |
| Spironolactone must be avoided with potassium rich foods. | | | | |  Yes |  No |  IDK |
| Cranberry juice can decrease effect of warfarin | | | | |  Yes |  No |  IDK |
| Patients on Warfarin should avoid foods like Spinach, broccoli in large quantities | | | | |  Yes |  No |  IDK |


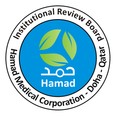
MRC-01-18-448 Validity: 16 06 2019 - 14 06 2020 E-stamped 20 Jun 2019

| Patient on NSAIDs should consume large amount of tea, coffee & chocolates | | | |  Yes | |  No |  IDK |
| --- | --- | --- | --- | --- | --- | --- | --- |
| Caffeine increases the risk of theophylline drug toxicity (like nervousness and tremor) | | | |  Yes | |  No |  IDK |
| Patient on L-thyroxin must avoid turnips, cauliflower, and cabbage | | | |  Yes | |  No |  IDK |
| Grape fruit interacts with different medications and may produce lethal side effect | | | |  Yes | |  No |  IDK |
| Meals rich in protein and fat can enhance levodopa absorption | | | |  Yes | |  No |  IDK |
| Esomeprazole absorption could be increased with fatty meals | | | |  Yes | |  No |  IDK |
| L-thyroxin requires the interruption of diet through feeding tube for 1-2 hours | | | |  Yes | |  No |  IDK |
| Phenytoin requires the interruption of diet through feeding tube for 1-2 hours | | | |  Yes | |  No |  IDK |
| Alendronate must be taken on empty stomach | | | |  Yes | |  No |  IDK |
| ***Timing*** | | | | | | | |
| Lansoprazole should be taken …………. meal | | |  Before | |  With/After | |  IDK |
| Glipizide, Rifafour^®^ should be taken ………. meal | | |  Before | |  With/After | |  IDK |
| NSAIDs, steroids are advised to be taken………. meal | | |  Before | |  With/After | |  IDK |
| Rivaroxaban dose ≥15 mg must be taken ………. meal | | |  Before | |  With/After | |  IDK |
| Thyroid hormones should be taken ………. meal | | |  Before | |  With/After | |  IDK |
| Metformin , gliclazide M/R should be taken ……..meal | | |  Before | |  With/After | |  IDK |
| Genvoya^®^ (Elvitegravir/cobicistat/tenofovir/emtricitabine) should be taken……. meal | | |  Before | |  With/After | |  IDK |
| Furosemide should be taken ………. meal | | |  Before | |  With/After | |  IDK |
| Cinacalcet should be taken …………. meal | | |  Before | |  With/After | |  IDK |
| **Believe, Attitude, and Practice about FDI** | | | | | | | |
| **Have you encountered any FDI before**   Yes  No | **Do you believe it’s necessary to know about FDI**   Yes  No | **Do you have any system to identify FDI in the work place**   Yes  No | | | | | |
| **How do you rate your knowledge about FDI**   Excellent .  Very good   Satisfactory  Poor | **Are you considering food interactions when scheduling the drug**   Yes  No | **Do you believe that each patient needs counseling about FDI**     Yes  No | | | | | |
| **How often do you educate patients about FDI**   Always  Sometimes   Never | **Who do you believe should educate patient about FDI**   Nurse  Pharmacist  Dietician .  Doctor | **Have you ever consult pharmacist about FDI**   Yes  No | | | | | |
| **What is your source of information if you want to check about FDI (***choose all apply***)**   Pharmacist  Dietician .  Drug leaflet  Online search engine  Other nurse  Book  Drug database | | | | | | | |
| **Which factor(s) do you believe can impact on FDI (***choose all apply***)**   Dosage form  Age  Health status  Body composition | | | | | | | |
| **How awareness about FDI can be improved MOSTLY among nurses:**   Conferences/seminar  Workshop  Online courses  Trusted references  Other: …………………… | | | | | | | |
| **Any other comment(s)** | | | | | | | |


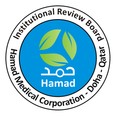
 ***Thanks***

MRC-01-18-448 Validity: 16 06 2019 - 14 06 2020 E-stamped 20 Jun 2019
